# Editorial: The Role of Inflammation in the Etiology and Treatment of Schizophrenia

**DOI:** 10.3389/fpsyt.2020.603296

**Published:** 2020-10-22

**Authors:** Ole Köhler-Forsberg, Norbert Müller, Belinda R. Lennox

**Affiliations:** ^1^Psychosis Research Unit, Aarhus University Hospital – Psychiatry, Aarhus, Denmark; ^2^Department for Affective Disorders, Aarhus University Hospital – Psychiatry, Aarhus, Denmark; ^3^Department of Clinical Medicine, Aarhus University, Aarhus, Denmark; ^4^Department of Psychiatry and Psychotherapy, Ludwig-Maximilians-University Munich, Munich, Germany; ^5^Department of Psychiatry, University of Oxford, Oxford, United Kingdom

**Keywords:** schizophrenia, inflammation, immunopsychiatry, anti-inflammatory, psychose, autoimmune psychosis

## Introduction

Schizophrenia is a severe mental disorder with heterogeneous clinical presentations and several psychological, social, and biological mechanisms. One potential biological mechanism, which has received increasing attention during the recent three decades, is that inflammatory processes may contribute to the etiology of schizophrenia and affect treatment response patterns. The inflammatory hypothesis is based on several observations, here among studies showing that (1) patients with schizophrenia have increased peripheral and central pro-inflammatory markers (1, 2), (2) genes coding for the immune system are more frequently expressed in patients with schizophrenia (3), (3) infections have been associated with an increased risk for subsequent development of schizophrenia (4), and (4) clinical trials have found treatment effects of anti-inflammatory medications on schizophrenia symptoms (5). The potential for this research is in defining an inflammatory subgroup and potentially leading to more tailored treatment possibilities for some patients, i.e., personalized medicine. However, before this can happen, several aspects of the interaction between inflammation and the development and course of schizophrenia need to be investigated and the potential for confounding factors need to be addressed, e.g., lifestyle, genetic, and disease-related factors. It is for example well-known that patients with schizophrenia have a more sedentary and unhealthier lifestyle, which may all contribute to an inflammatory profile.

The present Frontiers Psychiatry Research Topic “The Role of Inflammation in the Etiology and Treatment of Schizophrenia” represents a collection of 11 research papers approaching and describing different aspects of the inflammatory hypothesis in schizophrenia while at the same time putting the present knowledge into perspective and discussing future perspectives. The papers cover historical perspectives, preclinical studies, register-based studies, case reports, and reviews and give a broad, thorough and up-to-date overview of this field of psychiatric research.

The association between infection, inflammation, and psychosis is not a new observation. Kepińska et al. present the historical evidence including potential mechanisms for the association between influenza and psychosis with a specific focus on the Spanish Influenza Pandemic in 1918–1919. During active infection with the influenza virus, some otherwise healthy individuals developed psychiatric including psychotic symptoms which disappeared after recovery from the flu. This aspect is of particular current interest due to the suspected psychiatric symptoms caused by the ongoing Corona-virus disease (COVID-19) pandemic (6). The high comorbidity between schizophrenia and inflammatory diseases is further emphasized in a large UK database study, where Meier et al. investigated the bidirectional comorbidity between multiple sclerosis with schizophrenia and bipolar disorder. Whether inflammatory processes also may contribute to negative symptoms, some of the most disabling and difficult-to-treat symptoms of schizophrenia, is reviewed by Goldsmith and Rapaport. The authors also discuss the literature on depression and inflammation regarding certain aspects of negative symptoms and the potential of anti-inflammatory drugs for negative symptoms. The complex integrated pathways of the potential association between the inflammatory cascade and schizophrenia is emphasized in a study on 409 individuals with schizophrenia by Severance et al., indicating that gastrointestinal and endocrine abnormalities may contribute to inflammation in schizophrenia.

However, the clinical challenge is to identify those patients with a clinically relevant inflammatory state, for example those patients where an immune-related disease, e.g., autoimmune encephalitis, has led to the psychiatric symptoms. Two cases are presented in this Research Topic. Meixensberger et al. present the case of a 39-year-old female patient who developed an anti-NMDA-R encephalitis in 2009 with predominant severe catatonic symptoms. Anti-inflammatory treatment led to full recovery with some discrete symptoms. The authors discuss the treatment during the subsequent 10 years and the findings from the 10-year follow-up investigation. Endres et al. present the case of an 18-year-old male patient with autism spectrum disorder who developed a severe catatonic syndrome over 2.5 years. Most standard tests for autoimmune psychosis were negative, but the serum and CSF tissue-based assay revealed antineuronal autoantibodies against an unknown epitope, leading to the conclusion that the patient was most probably experiencing an autoimmune psychosis; Immunosuppressive treatment led to partial improvement. These cases highlight the importance of keeping autoimmune causes of psychosis in mind and emphasize the importance of having psychiatry as part of the multidisciplinary treatment of people with autoimmune encephalitis (7). The clinical challenges and opportunities to measure mild neuroinflammation in the individual patient are described in the review article by Bechter.

While immune-modulating drugs are the treatment of choice against autoimmune encephalitis, another interesting question is whether treatments targeting inflammation may help in treating patients with schizophrenia. Several antipsychotic drugs are available, but with varying efficacy and primarily effect on psychotic symptoms (8), while negative symptoms are very challenging to treat. Fond et al. review which cytokines may be best used to indicate inflammation in patients with schizophrenia, which anti-inflammatory therapies have been found to yield better treatment effects, and potential next steps to tailor anti-inflammatory therapies in schizophrenia. Lotter et al. performed a preclinical study to investigate the therapeutic effects of *Garcinia mangostana* Linn and one of its active constituents, α-mangostin, alone, and as adjunctive treatment with haloperidol on schizophrenia related bio-behavioral alterations in a maternal immune-activation rat model.

As for future directions, several aspects need to be investigated in order to explore whether the association between inflammation and schizophrenia represents causality or rather a confounded epiphenomenon. One important aspect is to explore whether the peripheral inflammation also translates to central nervous system (CNS) inflammation. Glial cells and particularly microglia, the resident CNS macrophages, might yield further clues to understand CNS inflammatory processes. Hanger et al. give an introduction to microglia, their role in brain development and present protocols to differentiate microglia from human induced pluripotent stem cells and thereby study these cells in more detail. As *in-vivo* studies of neuroinflammation are challenging, imaging techniques give the possibility of visualizing and quantifying the activity of glial inflammatory responses in the CNS. De Picker and Morrens present a critical review of studies on psychotic patients using Positron emission tomography with ligands targeting translocator protein 18 kDa (TSPO PET), a method aiming to measure microglial activity. The authors discuss five hypotheses which may explain the observed variability in TSPO PET findings.

In conclusion, the role of inflammation in the pathogenesis of schizophrenia represents an exciting and hopeful area of research, with the potential for different treatment approaches, addressing the cause of the disorder, rather than solely the symptoms. Whilst further work is required to firmly establish the causal role for inflammation in the development of psychosis, and to characterize the subgroup of patients that would benefit from immunotherapy approaches, we feel that we are approaching the point where there is a critical mass of evidence that inflammation is relevant for a proportion of patients, and this requires translation into clinical practice. We look forward to the time when psychotic disorders are investigated, diagnosed and treated according to their immunophenotype.

## Author Contributions

OK-F wrote the first draft of the manuscript. NM and BL provided critical revision of the manuscript and important intellectual contributions. All authors read and approved the submitted version.

## Conflict of Interest

The authors declare that the research was conducted in the absence of any commercial or financial relationships that could be construed as a potential conflict of interest.
